# Higher iron pearl millet (*Pennisetum glaucum* L.) provides more absorbable iron that is limited by increased polyphenolic content

**DOI:** 10.1186/1475-2891-14-11

**Published:** 2015-01-23

**Authors:** Elad Tako, Spenser M Reed, Jessica Budiman, Jonathan J Hart, Raymond P Glahn

**Affiliations:** USDA/ARS, Robert W. Holley Center for Agriculture and Health, Cornell University, Ithaca, NY 14853 USA; Division of Nutritional Sciences, Cornell University, Ithaca, NY 14853 USA; Department of Food Science, Cornell University, Ithaca, NY 14853 USA

**Keywords:** Pearl millet, Biofortification, Iron bioavailability, Polyphenols, In vitro digestion/Caco- 2 cell model, Broiler chicken

## Abstract

**Background:**

Our objective was to compare the capacity of iron (Fe) biofortified and standard pearl millet (*Pennisetum glaucum* L*.*) to deliver Fe for hemoglobin (Hb)-synthesis. Pearl millet (PM) is common in West-Africa and India, and is well adapted to growing areas characterized by drought, low-soil fertility, and high-temperature. Because of its tolerance to difficult growing conditions, it can be grown in areas where other cereal crops, such as maize, would not survive. It accounts for approximately 50% of the total world-production of millet. Given the widespread use of PM in areas of the world affected by Fe-deficiency, it is important to establish whether biofortified-PM can improve Fe-nutriture.

**Methods:**

Two isolines of PM, a low-Fe-control (“DG-9444”, *Low-Fe*) and biofortified (“ICTP-8203 Fe”,*High-Fe*) in Fe (26 μg and 85 μg-Fe/g, respectively) were used. PM-based diets were formulated to meet the nutrient requirements for the broiler (*Gallus-gallus*) except for Fe (Fe concentrations were 22.1±0.52 and 78.6±0.51 μg-Fe/g for the *Low-Fe* and *High-Fe* diets, respectively). For 6-weeks, Hb, feed-consumption and body-weight were measured (n = 12).

**Results:**

Improved Fe-status was observed in the *High-Fe* group, as suggested by total-Hb-Fe values (15.5±0.8 and 26.7±1.4 mg, *Low-Fe* and *High-Fe* respectively, P<0.05). DMT-1, DcytB, and ferroportin mRNA-expression was higher (P<0.05) and liver-ferritin was lower (P>0.05) in the *Low-Fe* group versus *High-Fe* group. *In-vitro* comparisons indicated that the High-Fe PM should provide more absorbable-Fe; however, the cell-ferritin values of the *in-vitro* bioassay were very low. Such low *in-vitro* values, and as previously demonstrated, indicate the presence of high-levels of polyphenolic-compounds or/and phytic-acid that inhibit Fe-absorption. LC/MS-analysis yielded 15 unique parent aglycone polyphenolic-compounds elevated in the *High-Fe* line, corresponding to *m/z* = 431.09.

**Conclusions:**

The *High-Fe* diet appeared to deliver more absorbable-Fe as evidenced by the increased Hb and Hb-Fe status. Results suggest that some PM varieties with higher Fe contents also contain elevated polyphenolic concentrations, which inhibit Fe-bioavailability. Our observations are important as these polyphenols-compounds represent potential targets which can perhaps be manipulated during the breeding process to yield improved dietary Fe-bioavailability. Therefore, the polyphenolic and phytate profiles of PM must be carefully evaluated in order to further improve the nutritional benefit of this crop.

## Introduction

The World Health Organization estimates that approximately one-third of worldwide infant deaths and one half in developing countries can be attributed to malnutrition. More specifically, iron (Fe) deficiency is the most common nutritional deficiency worldwide [1]. Fe deficiency is particularly widespread in low-income countries because of a general lack of consumption of animal products (which can promote non-heme iron absorption and contain highly bioavailable heme Fe) coupled with a high consumption of cereal grains and legumes replete with antinutrients (e.g., polyphenolic compounds and phytic acid) that are inhibitors of Fe bioavailability [2, 3].

Poor dietary quality is more often characterized by micronutrient deficiencies or reduced mineral bioavailability, than by insufficient energy intake [3, 4]. Diets with chronically poor Fe bioavailability which result in high prevalence of iron deficiency and anemia increase the risk of all-cause child mortalities and also may lead to many pathophysiological consequences including stunted growth, low birth weight, delayed mental development and motor functioning, and others [5–7]. Thus, a crucial step in alleviating Fe deficiency anemia is through understanding how specific dietary practices and components contribute to the Fe status in a particular region where Fe deficiency is prevalent.

Pearl millet (PM) is a resilient cereal crop, grown mostly in marginal environments in the arid and semi-arid tropical regions of Asia and Africa [8–10]. It is a major dietary constituent for peoples living in Western India and the Sahel region of the African continent, and is often served as a complementary food for infants and young children [11, 12]. For example, among the rural poor in India, PM intake can reach nearly 60% of all cereal grain consumption [8]. A major non-nutritional advantage to PM consumption is that it can be grown in areas with very limited rainfall, where crops such as maize or sorghum are very likely to fail during most growing seasons [8, 13]. As a well-adapted crop to growing areas characterized by drought, low soil fertility, and high temperature, it performs well in soils with high salinity or low pH [13, 14].

With regard to nutritional quality, PM is at least equivalent to maize and generally superior to sorghum in protein content/quality and metabolizable energy levels [10], as well as digestibility [15]. Furthermore, PM does not usually contain significant amounts of condensed polyphenols, such as the tannins commonly found in other staple crops such as sorghum, which can decrease digestibility [16]. PM grain is also rich in important micronutrients such as Fe and Zn, and has a more complete amino acid profile than maize or sorghum [15]. Taken in totality, these qualities make PM a major contributor of dietary protein, Fe, and Zn intake in a variety of rural populations in India and sub-Saharan Africa [10, 11].

Recently, conventional plant breeding at ICRISAT (International Crops Research Institute For the Semi-Arid Tropics, Andhra Oradesh, India) has developed biofortified PM containing up to 90 μg Fe/g PM, a substantial increase over standard PM containing 36-50 μg Fe/g PM [17]. A previous study that assessed biofortified PM line to deliver more absorbable Fe to young women indicated that consumption of the Fe biofortified PM increased the amount of absorbable Fe [18]. Another study in young children assessed the absorption of Fe and Zn from biofortified PM, and found that the concentrations of both Fe and Zn absorbed were more than adequate to meet the physiological requirements for these micronutrients [11].

However, an increase in Fe concentration in PM may not necessarily translate into a proportional increase in absorbed Fe since genotypes with high Fe concentrations may also have increased (or decreased) concentrations of Fe absorption inhibitors or enhancers [19]. Therefore, it is necessary to measure both the amount of bioavailable Fe and the concentration of Fe in these new iron-enhanced crops, as well as potential inhibitors (e.g., polyphenols) of Fe bioavailability [19, 20].

The *Gallus gallus* model has been used extensively for nutritional research and has shown to be an excellent animal to model Fe bioavailability [21], as chicks respond quickly to Fe malnutrition, and their micronutrient deficient phenotypes include poor Fe status, growth stunting, and organ hypertrophy. Further, this model agrees well with *in vitro Caco-2* cell results [19, 20, 22–26]. Hence, the objective of the current study was to compare the capacities of two pearl millet varieties to deliver Fe for Hb synthesis and to improve the Fe status of Fe deficient broiler chickens.

## Materials and methods

### Diets, animals and study design

The two pearl millet isolines used in the study were developed from a low Fe commercial variety for India (DG-9444, “*Low-Fe”*) and an introgressed, open pollinated variety line (ICTP 8203 Fe, “*High-Fe”*). Seed was multiplied in Andrha Pradesh, India under phosphorus fertilized, standard agronomic conditions and shipped to Ithaca, New York in sealed containers imported as grain.

Forty eight Cornish cross fertile broiler eggs were obtained from a commercial hatchery (Moyer’s chicks, Quakertown, PA). The eggs were incubated under optimal conditions at the Cornell University Animal Science poultry farm incubator. Upon hatching (hatchability rate was 93%), chicks were allocated into 2 treatment groups on the basis of body weight, gender and blood Hb concentration (aimed to ensure equal distribution between groups, n = 12): 1. *High-Fe*: 75% pearl millet diet (78 μg/g Fe); 2. *Low-Fe*: 75% pearl millet diet (22 μg/g Fe). Experimental diets had no supplemental Fe. Diets compositions are shown in Table 1.

**Table 1:** **Composition of the experimental diets** ^**1−3**^

Ingredient	***High-Fe***	***Low-Fe***
	(Biofortified)	(Standard)
	**g/kg (by formulation)**	
*High-Fe* Pearl millet (84.9 μg/g Fe)	750	−
*Low-Fe* Pearl Millet (25.9 μg/g Fe)	−	750
Skim milk, dry	100	100
DL- Methionine	2.5	2.5
Corn starch	47.5	47.5
Corn oil	30	30
Choline chloride	0.75	0.75
Vitamin/mineral premix (no Fe)	70	70
Total (g)	1000	1000
**Selected components**	**mean ± SEM, n = 5 (by analysis)**	
Dietary Fe concentration (μg/g)	78.6 ± 0.51^a^	22.1 ± 0.52^b^
Phytic Acid (μg/g)	9940 ± 1380^a^	10500 ± 230^a^
Phytate:Fe molar ratio^3^	10.7 ± 0.55^b^	40.2 ± 0.35^a^

^1^Vitamin and mineral premix provided/kg diet (330002 Chick vitamin mixture; 235001 Salt mix for chick diet; *Dyets* Inc. Bethlehem, PA).

^2^Iron concentrations in the diets were determined by an inductively-coupled argon-plasma/atomic emission spectrophotometer (ICAP 61E Thermal Jarrell Ash Trace Analyzer, Jarrell Ash Co. Franklin, MA) following wet ashing.

^3^Method for determining phytate is described in the materials and methods section.

^a,b^Within a row, means without a common letter are significantly different (P < 0.05).

Chicks were housed in a total-confinement building (1 chick per 0.5 m^2^ metal cage). Birds were under indoor controlled temperatures and were provided 16 h of light. Each cage was equipped with an automatic nipple drinker and manual self-feeder. All birds were given *ad libitum* access to water (Fe content was 0.379 ± 0.012 mg/L). Feed intakes were measured daily (as from day 1). Dietary Fe intake was calculated from feed intake and Fe concentration in the diets.

### Blood analysis and Hemoglobin (Hb) measurements

Blood samples were collected weekly from the wing vein (n = 12, ~100 μL) using micro-hematocrit heparinized capillary tubes (Fisher, Pittsburgh, PA). Samples were collected in the morning (starting at 08:00) following an 8 h overnight fast. The samples were analyzed for Hb concentration. Body weights and Hb concentrations were measured weekly.

Fe bioavailability was calculated as hemoglobin maintenance efficiency (HME) [21]:


Where Hb-Fe (index of Fe absorption) = total body Hb-Fe. Hb-Fe was calculated from Hb concentrations and estimates of blood volume based on body weight (a blood volume of 85 mL per kg body weight is assumed) [21]:


At the end of the experiment (day 42), birds were euthanized by CO_2_ exposure. The digestive tracts and livers were quickly removed (within 1 min post death) from the carcass and separated into various sections for tissue (duodenum and liver ~ 1-2 cm; ~2-3 g, respectively). The samples were immediately frozen in liquid nitrogen, and then stored in a −80°C freezer until analysis. All animal protocols were approved by the Cornell University Institutional Animal Care and Use Committee. Blood Hb concentrations were determined spectrophotometrically using the cyanmethemoglobin method (H7506-STD, Pointe Scientific Inc. Canton, MI) following the kit manufacturer’s instructions.

### Isolation of total RNA

Total RNA was extracted from 30 mg of duodenal (proximal duodenum, n = 12) and liver tissues (n = 12) using Qiagen RNeasy Mini Kit (Qiagen Inc., Valencia, CA) according to the manufacturer’s protocol. All steps were carried out under RNase free conditions. RNA was quantified by absorbance at 260–280 nm. Integrity of the 28S and 18S rRNA was verified by 1.5% agarose gel electrophoresis followed by ethidium bromide staining [19–26].

### DMT1, DcytB and ferroportin gene expression analysis

As previously described [19–26], PCR was carried out with primers chosen from the fragments of chicken (*Gallus gallus*) duodenal and hepatic tissues [Divalent Metal Transporter-1, DMT1 gene (GeneBank database; GI 206597489) (forward: 5’-AGC CGT TCA CCA CTT ATT TCG-3’; reverse: 5’-GGT CCA AAT AGG CGA TGC TC-3’), Duodenal Cytochrome B, DcytB gene (GI 20380692) (forward: 5’-GGC CGT GTT TGA GAA CCA CAA TGT T-3’; reverse: 5’-CGT TTG CAA TCA CGT TTC CAA AGA T-3’) and Ferroportin gene (GI 61098365) (forward: 5’-GAT GCA TTC TGA ACA ACC AAG GA’; reverse: 5’-GGA GAC TGG GTG GAC AAG AAC TC-3’)].Tissue-specific 18S rRNA was used to normalize the results [(GI 7262899) (forward: 5’- CGA TGC TCT TAA CTG AGT-3’; reverse: 5’-CAG CTT TGC AAC CAT ACT C-3’)]. All PCR products were separated by electrophoresis on 2% agarose gel, stained with ethidium bromide, and quantified using the Quantity One 1-D analysis software (Bio-Rad, Hercules, CA).

### *In-vitro*Fe bioavailability assessment

An *in vitro* digestion/*Caco-2* cell culture model [19] was used to assess *in vitro* Fe bioavailability. In this method, the cooked pearl millet samples and the formulated diets were subjected to simulated gastric and intestinal digestion. Exactly 0.5 g of the freeze-dried cooked pearl millet and diets samples were utilized for each replication of the *in vitro* digestion process.

### Harvesting of *Caco-2*cells for ferritin analysis

The protocols used in the ferritin and total protein contents analyses of *Caco-2* cells were similar to those previously described [19]. *Caco-2* cells synthesize ferritin in response to increases in intracellular Fe concentration. Therefore, we used the ratio of ferritin/total protein (expressed as ng ferritin/mg protein) as an index of the cellular Fe uptake. All glassware used in the sample preparation and analyses were acid washed.

### Ferritin and Fe in the liver, electrophoresis, staining and measurement of gels

Liver ferritin and liver Fe quantification were conducted as previously described [21]. The gels were scanned with Bio-Rad densitometer. Measurements of the bands were conducted using the Quantity-One 1-D analysis program (Bio-Rad, Hercules, CA). All samples (n = 6) were analyzed in duplicates (n = 6).

### Polyphenolic relative amounts in diets

A list of reported compounds was obtained by generation of high accuracy mass-to-charge (*m/z*) data derived from analysis of the PM samples using a UPLC/MS system and related software [19, 27]. From this *m/z* data, the METLIN database (METLIN, Scripps Center, La Jolla, CA) was used to identify a further list of potential flavonoid aglycones present in greater concentration in the *High-Fe* PM, and compiled in Table 2.

**Table 2:** **Aglycone of polyphenolic compounds corresponding to an** ***m/z*** **= 431.09 highly-enriched in the** ***High-Fe*** **PM**

Class	Compound	Putative ***in vitro***effect on Fe absorption/bioavailability	Citation
**Flavones**	Apigenin	↓	[28, 29]
	Baicalein	↓	[30, 31]
	Luteolin	↓	[28]
	Norwogonin	*	
	Scutellarein	*	
	5,7,2'-Trihydroxyflavone	*	
	7,3',4'-Trihydroxyflavone	*	
	7,3',4',5'-Tetrahydroxyflavone	*	
**Flavonol**	Galangin	↓	[32]
	Kaempferol	↓	[28]
**Isoflavones**	Dihydrodaidzein	↓	[28]
	Genistein	↓	[29, 33]
	Trihydroxyisoflavone	*	
	6,7,4’-trihydroxyisoflavone	*	
**Anthocyanins**	Pelargonidin	↓	[34]

*As of the writing of this paper, no data on the putative effects of these compounds relating to Fe absorption/ bioavailability exist.

↓ Decrease of Fe bioavailability/absorption *in vitro*.

### Polyphenol extraction

As previously described [19, 27], to one gram of ground PM material, 5 mL of methanol:water (50:50) was added. The slurry was vortexed for one minute, placed in a sonication water bath for 10 minutes, vortexed again for one minute, and centrifuged at 4000 × g for 15 min. The supernatant was filtered with a 0.45 μm syringe filter and stored for later use in a −20°C freezer.

### LC/MS analysis

As previously described [19, 27], Extracts were analyzed by LC-MS with an Acquity UPLC coupled to a Xevo G2 QTof spectrometer (Waters Corp. Milford, MA). For LC analysis, 5 μL samples of extract were injected and passed through a HSS C18 1.8 μm 2.1 × 100 mm column (Waters) at 0.4 mL/min. The mobile phase consisted of water with 0.1% formic acid (solvent A) and acetonitrile with 0.1% formic acid (solvent B). Polyphenols were eluted using linear gradients of 2.4 to 20% B in 2.5 min, 20 to 40% B in 0.5 min, 40 to 52% B in 2 min, 52 to 95% B in 0.5 min, 95 to 2.4% B in 1 min, and a 0.5 min hold at 2.4% B. ESI mass spectrometry was performed in positive ionization mode with a scan speed of 5/s in the mass range from 50 to 1200 Da. Lock-mass correction was used, with leucine-enkephalin as the external lock-mass standard. Instrumentation and data acquisition were controlled by MassLynx (Waters Corp., Milford, MA) software. Eluted compounds were marked by mass (m/z) and relative abundance using MarkerLynx (Waters Corp., Milford, MA) software. Potential polyphenol identities of individual masses were obtained by reference to METLIN database (Scripps Center for Metabolomics).

### Determination of phytic acid concentration in the diet samples

Dietary phytic acid (phytate)/total phosphorus was measured as phosphorus released by phytase and alkaline phosphatase, following the kit manufacturer’s instructions (n = 5) (K-PHYT 12/12, Megazyme International, Ireland).

### Statistical analysis

Results were analyzed by ANOVA using the general linear models procedure of SAS software (SAS Institute Inc. Cary, NC). Differences between treatments were compared by Tukey’s test and values were considered statistically different at P < 0.05 (values in the text are means ± SEM).

## Results

### Growth rates, Hb, Hb-Fe and HME

**Figure 1:**
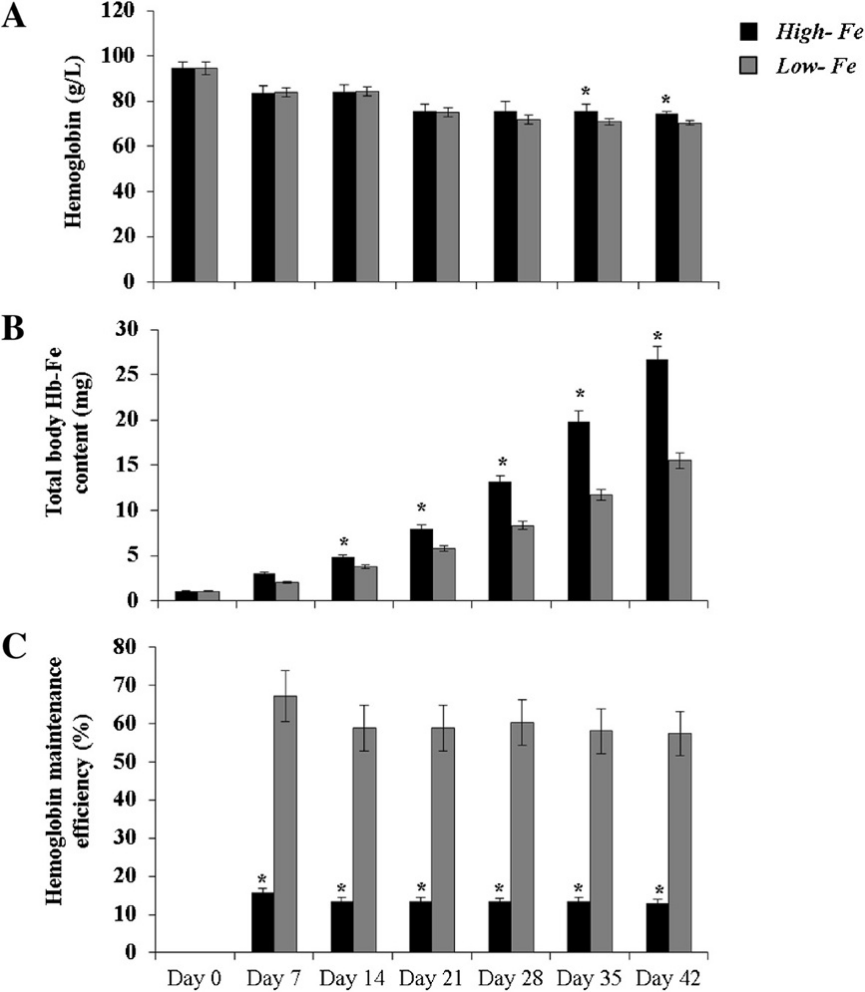
**Iron status parameters of chicken fed the tested diets from days 0- 42** ^**1**^ **. (A)** Hb (g/L), **(B)** Total body Hb-Fe content (mg), and **(C)** % HME. ^1^Values are mean daily feed intakes for the 7 days preceding the day designated in the column heading (n=12).

There were no significant differences in feed intakes at any time throughout the study. However, Fe intakes were consistently higher in the *High-Fe* group versus *Low-Fe* group. As from day 14 of the study, body weights were higher (P < 0.05) in the *High-Fe* group versus *Low-Fe* group. Also, as from day 35 of the study, Hb concentrations were higher (P < 0.05) in the *High-Fe* group versus *Low-Fe* group (Figure 1). The increase in total body Hb-Fe from day 14 until study conclusion was significantly greater in the *High-Fe* group versus *Low-Fe* group (25.6 ± 1.4 mg and 14.4 ± 0.8 mg, respectively, P < 0.05, Figure 1). HME was significantly different between groups at all-time points, with a higher percent obtained in the bird group receiving the standard PM diet (*Low-Fe*, n = 6, P < 0.05).

### Gene expression of iron transporters (DMT-1, Ferroportin) and DcytB in the duodenum

**Figure 2:**
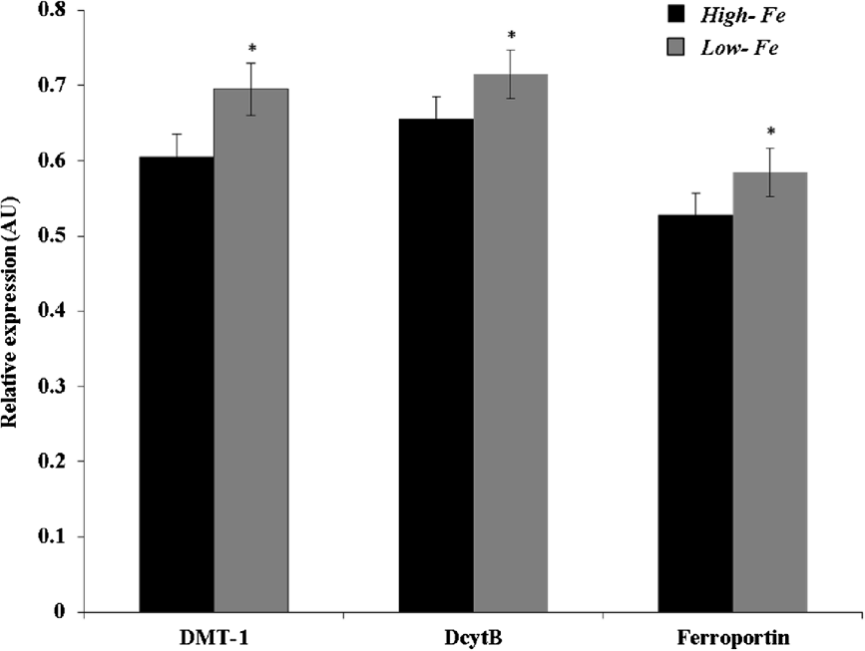
**Duodenal mRNA expression of DMT-1, DcytB, and ferroportin on day 42.** ^1^Changes in mRNA expression are shown relative to expression of 18S rRNA in arbitrary units (AU, n = 12, P < 0.05).

Gene expression analysis of duodenal DMT-1, Ferroportin and DcytB, with results reported relative to 18S rRNA, revealed increased mRNA expression of DMT-1, Ferroportin, and DcytB in the *Low-Fe* group compared to the *High-Fe* group (Figure 2) (n = 6, P < 0.05).

### *Caco-2*cell ferritin protein formation

Ferritin concentrations were significantly higher in cells exposed to the *High-Fe* diet versus the *Low-Fe* diet, as well as higher in cells exposed to the *High-Fe* PM versus *Low-Fe* PM only (P < 0.05, n = 6, Table 3).

**Table 3:** **Ferritin concentrations in** ***Caco-2*** **cells exposed to samples of PM only and PM-based diets**

Tested sample ^1^	Ferritin/cell protein (ng/mg)
Cell Baseline^2^	1.54d^e^ ± 0.12
FeCl_3_	58.69^b^ ± 2.29
FeCl_3_ + Ascorbic Acid	364.95^a^ ± 19.55
*Low-Fe* PM only	1.22^e^ ± 0.05
*High-Fe* PM only	2.61^c^ ± 0.36
*Low-Fe* PM-based diet	1.47^de^ ± 0.27
*High-Fe* PM- based diet	2.46^c^ ± 0.13

^1^ *Caco-2* bioassay procedures and preparation of the digested samples are described in the materials and methods sections (mean ± SEM).

^2^Cells were exposed to only MEM (minimal essential media) without added food digests and Fe (n = 6).

^a-e^ Within a column, means without a common letter are significantly different (P < 0.05).

### Ferritin and Fe in the liver

The avian ferritins corresponded to a weight of approximately 470 to 500 kDa [21]. No significant differences in liver Fe or liver ferritin concentrations (with liver specimens collected on day 42) were measured between the treatment groups (n = 6, P > 0.05, Table 4).

**Table 4:** **Ferritin protein and the iron concentration in the liver** ^**1**^

Treatment diet	Ferritin (μg/g wet weight)	Iron (μg/g wet weight)	Iron/Ferritin (μmol)
*High-Fe*	285 ± 8.5^a^	25.2 ± 3.9^a^	34.5 ± 3.5^a^
*Low-Fe*	277 ± 7.1^a^	19.3 ± 2.7^a^	29.7 ± 5.3^a^

^1^Atomic mass for iron used for calculations defined as 55.8 g/mol.

^a^Within a column, means with a common letter are not significantly different (n = 12, P > 0.05, mean ± SEM).

### Polyphenolic relative amounts in the diets samples

Phenolic analysis [19, 27] of the PM samples detected three specific mass-to-charge ratios (*m/z*), one of which significantly higher in the *High-Fe* (biofortified) PM variety (AU, P < 0.05). The elevated mass (*m/z =* 431.09) corresponds to 15 possible candidate glycosylated phenolic compounds. The aglucones of these compounds, as well as their purported effect on Fe absorption and bioavailability [28–34], can be found in Table 2.

### Phytate concentration and Phytate:Fe molar ratios in the diet samples

No significant differences in phytate concentration were measured between *High-Fe* and *Low-Fe* diets (n = 5, P > 0.05). Dietary phytate concentrations (as inositol hexaphosphate, IP6) are shown in Table 1. The concentrations of phytic acid (IP_1→6_) and Fe in the diets were used to calculate the phytate to Fe molar ratios. However, as expected, the ratios of phytate:Fe significantly differ between diets (40.2 ± 0.35 and 10.7 ± 0.55 for the *Low-Fe* and *High-Fe* PM diets, respectively, n = 5, P > 0.05, Table 1).

## Discussion

PM is a pervasive and nutritious grain harvested in many parts of the world; it is common primarily in West Africa and the Indian subcontinent, where micronutrient deficiencies are rampant [8]. It is an unusually hardy food crop, and consequently there is a progressive increase in the use of these grains as a major food staple, especially among subsistence farmers and the rural poor in large areas of India and sub-Saharan Africa [8, 35]. In terms of biofortification, target levels for PM Fe concentration have been set at nearly 77 μg/g or higher, which should likely represent a 30–40 μg/g differential from the more typical PM Fe levels [17]. In the present study, the differential in Fe content between the two PM lines was 56 μg/g, thus confidence was high going into the study that a nutritional benefit would be observed. In addition, it was recently demonstrated that Fe biofortified PM improved Fe status in Indian school children, and the authors concluded that dietary supplementation with Fe biofortified PM for six months significantly resolved Fe deficiency [36]. Hence, the objectives of the current study were to assess the capacity of a Fe-biofortified PM line to provide bioavailable Fe for Hb synthesis, as well as to establish a polyphenolic profile of the PM variety.

The *in vitro* results showing increased ferritin concentrations in *Caco-2* cells exemplify that the *High-Fe* (biofortified) PM does in fact provide additional, absorbable Fe. However, the assay also suggests that the bioavailability is relatively low compared to other foods, as the ferritin values are only slightly higher than the “baseline” conditions. As was previously demonstrated, such low values are typical of an inhibitory effect by polyphenols [19, 20, 22, 27]. Although hepatic ferritin and Fe concentrations were not significantly different between groups, increases in Hb (on days 35 through 42 in the *High-Fe*) and total body Hb-Fe (higher as from day 14 in the *High-Fe*) indicate birds receiving the *High-Fe* diet had moderately higher Fe available for Hb synthesis. Further, % HME was significantly elevated at all time points in the *Low-Fe* indicating an adaptive response (e.g., a relative up-regulation of absorption) to less absorbable dietary Fe. In addition, significant differences in duodenal mRNA abundance of DMT-1, DcytB and ferroportin were obtained between groups, with a higher relative mRNA expression of all three genes in the *Low-Fe* group. Similar to previous observations [19, 20, 24, 25], these results suggest, again, a compensatory, or adaptative, mechanism in the *Low-Fe* group due to a relative deficiency of absorbable Fe in the diet. In totality, however, these results suggest that the *High-Fe* PM diet provided more absorbable Fe to the birds, and thus yielded an improved Fe status throughout the duration of the study.

The interference of Fe uptake, relative to control diets high in bioavailable Fe, reflected in the *Caco-2* cell results (Table 3) is indicative of the strong inhibitory effect that so-called anti-nutrients (e.g., polyphenolic compounds) have on Fe bioavailability [37]. Although, for example, differences in ferritin concentration in *Caco-2* cells exposed to the *High-Fe* PM diet versus the *Low-Fe* PM diet were obtained, however, this relatively higher amount of ferritin (in the *High-Fe* PM) is not proportional to the significantly increased Fe content in the biofortified *High-Fe* PM. Although the *High-Fe* PM contained a greater Fe concentration than did the *Low-Fe* PM, concentration of polyphenolic compounds, known inhibitors of Fe bioavailability [38], also increased. Therefore, as part of the breeding process, it is incumbent upon researchers to assess the polyphenolic profile of the biofortified crop in question, since these chemicals have significant effects on Fe absorption and bioavailability in a variety of cell culture, animal, and human models [37–39].

From our LC/MS analysis, we determined a *m/z* ratio of 431.09 corresponding to 15 unique parent polyphenolic aglycones, significantly elevated in the *High-Fe* PM compared to the *Low-Fe* PM. The plant metabolites identified belong to chemical families including flavones, flavonols, isoflavones, and anthocyanins, many of which have been shown to inhibit Fe absorption [28–34], [Table 2] either by direct mineral chelation and Fe efflux or, in the case of the phytoestrogen isoflavones, by modulating membrane Fe receptor expression and thus affecting Fe homeostasis [33]. For example, [31] elucidated antioxidant effects of baicalein through Fe-binding in a physiologically-relevant *in vitro* model. It was determined that baicalein bound Fe^2+^ more strongly than ferrozine, a well-known Fe^2+^ chelator. Our results are consistent with others [40–42] who have found a variety of phenolic and polyphenolic compounds, namely kaempferol, luteolin, and apigenin, in different varieties of millet (mainly *E. coracana*, a utricles millet). For a detailed review of relevant phenolic compounds found in millet, please see [43] and [44].

Indeed the purpose of the current study was to assess Fe bioavailability in biofortified PM, however, future research is certainly needed to elucidate what, if any, affects these other compounds mentioned in Table 2 have on mineral absorption and bioavailability. In light of the significant biological effects these polyphenols have in modulating many aspects of health and chronic disease [45], a goal of future research should be to identify and modulate concentration of specific families, and perhaps individual compounds, which display Fe inhibitory properties. Using this tailored, individualistic approach, the health-promoting properties of these compounds may remain largely intact in PM and other crops, while the effects of Fe inhibition suppressed. In India alone, about 50 million people rely upon PM as a major source of their dietary energy. Its tolerance to drought, heat and soil salinity and its high water use efficiency makes it a climate-smart crop. In addition, given its high protein and mineral content (especially Fe), and high dietary fiber, the area under PM cultivation is expected to increase, including its adoption in non-traditional growing environments [2, 8–13]. Hence, we suggest continued research using the *in vitro*/ *Caco-2* cell and *Gallus gallus* models as guiding tools to further investigate these effects.

## Conclusion

This study provides evidence that increasing Fe concentration in biofortified PM by nearly 60 μg/g provides modest, yet noticeable, increases in bioavailable Fe *in vitro* and improved Fe status *in vivo*. Concurrent increases in polyphenolic compounds, inhibitors of Fe utilization, in the biofortified PM suggest that these compounds must be considered when using high- Fe PM lines to improve the Fe status of at-risk populations. Future feeding trials must continue to characterize the polyphenolic and phytate profiles of PM, and evaluate the effects such compounds have on Fe absorption and bioavailability. Modification of the PM polyphenol profile may be a means to improve Fe bioavailability in PM. We conclude that PM is a promising vehicle for increasing intakes of bioavailable Fe.

## Abbreviations

PM: Pearl millet
Fe: Iron
Zn: Zinc
Hb: Hemoglobin
Hb- Fe: Hemoglobin iron
HME: Hemoglobin maintenance efficiency
BW: Body weight
ICRISAT: International Crops Research Institute for the Semi-Arid tropics
LC/MS: Liquid Chromatography/ Mass Spectrometry
ESI: Electron Mass Spectrometry
UPLC: Ultra Performance Liquid Chromatography
PCR: Polymerase chain reaction
DMT-1: Divalent metal transporter 1
DcytB: Duodenal cytochrome B
Da: Dalton
MEM: Minimum essential media
AU: Arbitrary Units.
